# The Risk of the Development of Secondary Post-Traumatic Stress Disorder among Pediatric Health Care Providers: A Systematic Review

**DOI:** 10.3390/reports6010009

**Published:** 2023-02-23

**Authors:** Nikolaos Rigas, Alexandra Soldatou, Maria Dagla, Christina Nanou, Evangelia Antoniou

**Affiliations:** 1 Department of Midwifery, University of West Attica, 122 43 Egaleo, Greece; 2 Faculty of Medicine, National and Kapodistrian University of Athens, 157 72 Athens, Greece

**Keywords:** PTSD, secondary PTSD, burnout, pediatric patients, pediatric health care providers

## Abstract

Background: Secondary PTSD is defined as the natural, consequential behaviors and emotions that result from knowledge about a traumatic event experienced by a significant other deriving from working with suffering individuals. Pediatric health providers with symptoms of PTSD report problems with relationships and general life dissatisfaction as well as and anxiety, depression and burnout syndrome. Aim: The aim of this systematic review was to estimate the risk of developing secondary PTSD among pediatric health care providers as well as all additional contributing factors. In more detail, we investigated the PTSD symptomatology between pediatric health care providers and the extent to which parameters such as the job, gender, department and other factors had an impact on the mental health status of pediatric health care providers. Methodology: We searched all published English papers in PubMed, Google Scholar and the Cochrane Library from September to November 2022. We excluded reviews, systematic reviews and meta-analyses as well as letters to editors. From a total of 748 papers, we included only 12 research articles that met the admission criteria. Results: According to our results, the prevalence of secondary PTSD ranges from 13% to 94%. Burnout, nurses, the female gender, intensive care units, past traumatic life events and a psychiatric history identified as factors contributing to the development of secondary PTSD. Conclusions: Health policy-makers should take the specificity in the working environment of the pediatric sector seriously into consideration, especially emergency departments.

## 1. Introduction

Post-traumatic stress disorder (PTSD) has been recognized for centuries as a stress reaction, but only in 1980 was it accepted as a clinical diagnosis [1]. According to the American Psychiatric Association [2], PTSD is a psychiatric disorder that can occur in people who have experienced or witnessed a traumatic events such as natural disasters, serious accidents, terrorist acts, war/combat or rape, or who have been threatened with death, sexual violence or serious injury. The individual re-experiences the event, avoids the stimuli associated with the traumatizing event and experiences increased arousal.

PTSD can occur in all people of any ethnicity, nationality or culture and at any age. More than half of the adult population has been exposed to a severe stressor at some point during the course of their lives [3]. PTSD affects approximately 3.5% of US adults every year and it is estimated that almost one in eleven people will be diagnosed with PTSD in their lifetime [4]. It is estimated that 1in 11 people of all ages will be diagnosed with PTSD in their life [3]. However, women are twice as likely as men to have PTSD because certain types of trauma such as sexual assault are more common among them [5]. The prevalence of PTSD in the USA has been reported to be around 10% for women and 5% for men [6].

It is possible for almost everyone to develop a post-traumatic stress reaction shortly after being exposed to severe stressors. Most stress reactions usually diminish in a few days, weeks or months without any intervention. In several cases of those exposed, the outcome has been increased resilience, acceptance and post-traumatic growth [7]. The risk of developing PTSD depends on several factors such as the nature of the critical incident, the individual’s personality, the life history or the events that occurred in the aftermath of the trauma [8]. In many cases, a co-morbidity between PTSD and disorders such as depression, anxiety and substance abuse has been reported [7]. However, social and emotional support seems to be protective against the development of PTSD [9]. The most stressful jobs are the ones focusing on human health care, especially pediatric health care. Consequently, work stress can affect the physical and emotional well-being of employees by reducing their efficiency; at the same time, it has a negative impact on their social quality of life [10]. Several studies on the secondary traumatization of health care professionals have been published so far. Secondary PTSD is defined as the natural, consequential behaviors and emotions that result from knowledge about a traumatic event experienced by a significant other deriving from working with suffering individuals [11]. Individuals with evidence of secondary PTSD have PTSD-like symptoms, as determined by the Post-Traumatic Stress Scale [12] that investigates symptoms in the previous week. However, according to the *Diagnostic and Statistical Manual of Mental Disorders: DSM-5* [2], PTSD can also be caused after workplace exposure; thus, the PCL-5 scale is considered to be appropriate [13]. Much of the existing literature has focused on secondary PTSD rather than PTSD among pediatric health providers. Overcoming these differences and the effect of daily exposure to psychological trauma could have a significant impact on the well-being of each worker and, therefore, on the professional care of children. Symptoms of secondary PTSD in pediatric health providers include thinking of patient situations, avoiding situations that remind them of the workplace with distressed patients and feelings of arousal such as jumpiness or sleeping disturbances with a traumatized patient [12].

Plenty of children are presented for medical care (from illness and injury due to traumatic events such as violence and natural disasters) and medical professionals and support staff experience multiple trauma symptoms as they face life-threatening conditions or live in abusive environments. This continuous contact with a traumatized population brings to light the issue of secondary traumatization. Health providers with symptoms of PTSD report problems with relationships and general life dissatisfaction as well as anxiety, depression and burnout syndrome [14,15]. These diminish the staff morale, productivity and quality of care and increase the danger of medication errors, patient falls and other tools of patient morbidity [16,17,18]. Situations involving children are often considered to have a greater psychological impact; thus, pediatric workers may be at a particular risk of PTSD [11]. The literature has proven that PICUs and emergency departments are considered to be risk factors for the development of PTSD, given the high morbidity and mortality from which they are characterized [15,19]. In particular, intensive care units are stressful sources not only for patients and their families, but also for their staff; there is a proportionately greater amount of work stress than most professions [16]. However, the prolonged professional stress of professionals usually causes emotional exhaustion. Symptoms of depression, high rates of sickness absence, substance use (drugs or alcohol) or chronic physical pain have also been reported among unit staff. It is noteworthy that a large percentage of doctors (approximately 400) die from suicide each year, twice the rate of the general population [17]. Emergency departments are high-stress workplaces, similar to intensive care units. The staff who work there care for patients with trauma and life-threatening conditions such as strokes, heart attacks, burns, traffic accidents and violent accidents [18]. In addition, staff often have to provide care from one patient to another, with no time to process stressful events or engage in their own self-care. At the same time, they appear to have the highest rate of physical assaults of any department in the hospital [19]. Therefore, caring for dying patients is a daily routine for intensive care unit and emergency room staff. In addition to the above factors, it appears that perceived non-job satisfaction, burnout, compassion fatigue and the female gender [20] significantly contribute to the development of post-traumatic symptoms [11]. Given that managing the above stressful environments can be even more complex for providers when treating and caring for pediatric patients because the loss of a child is an extremely tragic event, we hypothesized that pediatric health professionals face a high risk of developing post-traumatic symptomatology. Thus, the aim of this systematic review was to estimate the risk of developing secondary PTSD among pediatric health care providers as well as all additional contributing factors.

## 2. Methods

The purpose of this study was to investigate the risk of developing PTSD among pediatric health care providers. In more detail, we investigated the PTSD symptomatology between pediatric health care providers and the extent to which parameters such as the job, gender, department and other factors had an impact on the mental health status of pediatric health care providers. To achieve the above objective, a systematic review of relevant studies was carried out based on Google Scholar, PubMed/Medline Cochrane Library and Scopus.

### 2.1. Eligibility Criteria

This study had specific inclusion and exclusion criteria. For example, we included research articles that investigated the relationship between pediatric health care providers and PTSD or secondary traumatization. This study followed the guidance of the Preferred Reporting Items for Systematic Reviews and Meta-Analyses (PRISMA) [21], the PROSPERO Systematic Review registration number: 401443. However, we excluded papers that were review articles such as reviews, systematic reviews, meta-analyses and letters to editors. We included original research articles (cross-sectional and cohort studies). We included health care providers who did not work with pediatric patients and did not report data on PTSD.

### 2.2. Search Strategy

**Figure 1:**
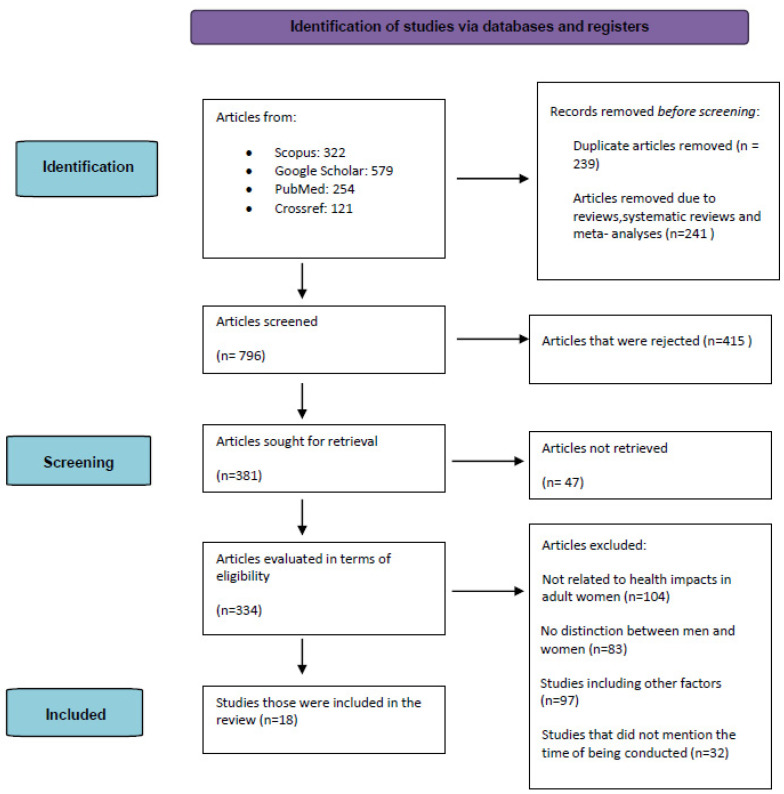
Flow chart of the included studies.

We searched all published English papers in PubMed, Google Scholar, the Cochrane Library and Scopus from September to November 2022. The years of publication ranged from 1999 to 2021.The terms used were: ((((((((Pediatric health care providers) OR (pediatric health care professionals)) OR (pediatric caregivers)) AND (secondary PTSD))) OR (PTSD)) OR (secondary traumatization)) OR (exposure to work related trauma)) OR (mental health). Firstly, we evaluated the titles and the abstracts. The full texts of the paper were then evaluated again using the eligibility inclusion and exclusion criteria. The first search found 748 papers. After we rejected double studies, other titles and papers that did not have a text, 141 papers remained to be evaluated. In addition, we rejected 123 papers due to them being reviews, systematic reviews and meta-analyses; 6 papers were written in a language other than English. Finally, 12 studies were included in the review (Figure 1).

### 2.3. Methodological Quality Evaluation of the Studies

The quality assessment tool for observational cohort and cross-sectional studies [22] was used to assess the quality of the studies (Table 1). Each question of the tool can be answered with “Yes”, “No” or “Not applicable”, investigating whether the criteria were met foreach paper. In more detail, the criteria consisted of 14 questions, which investigated: (1) the clarity of the research question; (2) the type of the study population; (3) the participation rate of the study population; (4) the predetermined inclusion/exclusion criteria of the participants; (5) the justification of the sample size; (6) if the exposure was measured before the outcome; (7) if there was sufficient time between the exposure and the outcome; (8) if the study examined different levels of exposure depending on the outcome; (9) the clearly defined measures of exposure; (10) if the exposure was assessed more than once over time; (11) clarity of the outcome measures; (12) whether the outcome assessors were blinded to the exposure status; (13) the sample loss to follow-up; and (14) the confounding variables that were measured and statistically adjusted. However, this tool was not used to derive an overall score, but mainly to examine the overall quality of a study and the risk of bias.

## 3. Results

All studies were cross-sectional and the majority of these took place in the USA (Table 2). All articles provided data on the exposure of pediatric health care providers to work-related trauma. Trauma exposure occurred in pediatric hospitals, pediatric intensive care units, neonatal intensive care units, pediatric units, pediatric trauma services, protective services and pediatric emergency departments.

Diagnostic tools were also used on all health care providers for the diagnosis of PTSD. We also extracted information on the additional risk factors that led to secondary PTSD.

According to our results, the prevalence of secondary PTSD ranged from 13% to 94%. The majority of studies mainly included nurses [11,19,27,28,29,30,31,32,33,34] and physicians [27,28,34]. Only one study included child protective workers [23] and three studies investigated secondary PTSD [19,34,35]; the other studies additionally examined burnout [11,27,28,29,30,31,32,34], compassion fatigue [27,28,29,30] and other outcomes relevant to a secondary trauma such as distress, depression, phobic anxiety, paranoid ideation and psychoticism symptoms [23]. However, the study of Beaudoin [32] did not specify the specialty of the primary caregivers. They also did not specify the research methodology they followed.

We identified several additional factors that could contribute to the development of secondary PTSD or PTSD. One study [23] showed that being assaulted or the threat of being assaulted on the job, the female gender and working more than 40 h a week affected the appearance of PTSD. Another study [24] reported that several years in the direct care of pediatric patients took a psychological toll on the health provider. On the other hand, two studies reported that fewer years of work was a stronger factor [29,30]. Higher rates of affectively mediated empathy were also associated with secondary PTSD according to the study of Robins et al. [24]. This was in contrast to the research of Ratrout et al. [20], which showed that lower rates of empathy were largely responsible for secondary PTSD symptoms. In addition, the job of nursing was considered to be a strong risk factor in two studies [27,31] where as one study identified the job of doctors [29]. A strong correlation was also observed between PTSD symptoms and burnout as well as low job satisfaction according to the study of Sekol et al. [26]. Working in a PICU was also more stressful than working in other pediatric departments; thus, a PICU was considered to be an aggravating factor [31,32]. Finally, a history of traumas [20], anxiety, depression [11], drinking alcohol [29] and less a problem-focused coping style [31] in pediatric care providers appeared to be vulnerability factors for the development of secondary PTSD.

**Table 2:** Studies included in the review.

Author/Year	Design	N	Data	Exposure	Instruments	Outcomes	Additional Risk Factors
Cornille 1999 [23] USA	Cross-sectional study	183 child protective workers	Child protective service (CPS) in a southern state	Child abuse trauma	1. Brief Symptom Inventory (BSI) [33] 2. Impact of Event Scale-Revised (IES-R) [34]	1. Secondary PTSD 2. Distress 3. Depression 4. Phobic anxiety 5. Paranoid ideation 6. Psychoticism symptoms	1. Assault or threat of assault on the job 2. Female gender 3. Work hours > 40 per week
Robins 2009 [24] USA	Cross-sectional study	314 health care professionals (86 physicians, 136 nurses, 43 mental health practitioners and 49 allied health)	The Children’s Hospital of Philadelphia	Exposure to secondary trauma	1. Interpersonal Reactivity Index (IRI) [35] 2. Spiritual Involvement Beliefs Scale (SIBS) [36] 3. Brief COPE [37] 4. Compassion Satisfaction and Fatigue Test (CSFT) [38]	1. Secondary PTSD 2. Compassion fatigue 3. Burnout	1. Several years in direct care 2. Higher rates of affectively mediated empathy 3. Nurses
Meadors 2010 [25] USA	Cross-sectional study	167 pediatric health care providers (nurses, chaplains and physicians)	Pediatric intensive care unit (PICU), neonatal intensive care unit (NICU) and/or pediatric unit (PEDS), Carolina	Exposure to secondary trauma	1. The Impact of Event Scale [34] 2. Professional Quality of Life Scale, version 5 (ProQOL-V5) [39]	1. Secondary PTSD 2. PTSD 3. Burnout 4. Compassion fatigue	N/A
Czaja 2011 [11] USA	Cross-sectional study	173 nurses of general medical, surgical and oncology wards as well as the pediatric intensive care unit and emergency room	Children’s hospital with a wide referral base and level 1 trauma services	Exposure to secondary trauma	1. The Post-Traumatic Diagnostic Scale (PDS) [40] 2. The Hospital Anxiety and Depression Scale (HADS) [41] 3. The Maslach Burnout Inventory (MBI) [42]	1. PTSD 2. Burnout	1. Anxiety 2. Depression
Sekol 2014 [26] USA	Cross-sectional study	240 registered nurses working in surgical, medical, critical care and hematology/oncology units	Tertiary acute care pediatric hospital, California	Exposure to secondary trauma	1. Professional Quality of Life Scale, version 5 (ProQOL-V5) [39] 2. Brief Index of Affective Job Satisfaction (BIAJS) [43]	1. Secondary PTSD 2. Burnout 3. Compassion satisfaction	1. 5–9 years of experience in the surgical unit 2. High burnout levels 3. Low job satisfaction
Berger 2015 [27] USA	Cross-sectional study	239 registered nurses working in pediatric units	A system of 5 hospitals that included an urban pediatric tertiary care teaching hospital	Exposure to secondary trauma	1. Professional Quality of Life Scale, version 5 (ProQOL-V5) [39]	1. Secondary PTSD 2. Burnout 3. Compassion satisfaction	1. Caucasian 2. Work experience ≤ 5 years
Branch 2015 [28] USA	Cross-sectional study	296 staff nurses, advanced practice nurses, social workers, respiratory therapists, physical therapists, occupational therapists, psychologists and child life therapists	St. Louis Children’s Hospital	Exposure to secondary trauma	1. Professional Quality of Life Scale, version 5 (ProQOL-V5) [39]	1. Secondary PTSD 2. Burnout 3. Compassion satisfaction	1. Nurses who work in the PICU
Colville 2017 [29]	Cross-sectional study	195 PICU staff and 164 ICU staff	3 adult ICUs and 4 PICUs	Exposure to secondary trauma	1. Brief Resilience Scale [44] 2. Maslach Burnout Inventory [42] 3. Trauma Screening Questionnaire [5] 4. Hospital Anxiety and Depression Scale [41]	1. Secondary PTSD 2. Burnout	1. Work in PICU 2. Doctors 3. Drink alcohol
Kellogg 2018 [30] USA	Cross-sectional study	338 certified pediatric nurses	All pediatric nurses	Exposure to secondary trauma	1. Secondary Traumatic Stress Scale [12] 2. Brief COPE [45] 3. Marlowe–Crowne Social Desirability Short Form [46]	1. Secondary PTSD	N/A
Rodríguez-Rey 2019 [31] Spain	Cross-sectional study	298 PICU staff members (physicians, nurses and nursing assistants) and 189 professionals working in non-critical pediatric units (physicians, nurses and nursing assistants)	9 hospitals	Exposure to secondary trauma	1. Brief Resilience Scale [44] 2. Coping Strategies Questionnaire for healthcare providers [47] 3. Maslach Burnout Inventory [42] 4. Trauma Screening Questionnaire [48]	1. Secondary PTSD 2. Burnout	1. Individuals who used a problem-focused coping style less
Ratrout 2020 [20] Jordan	Cross-sectional study	202 nurses	8 emergency departments in Jordan	Exposure to secondary trauma	1. Secondary Traumatic Stress Scale [12] 2. Life Events Checklist, Fifth Version (LEC-5) [49] 3. Toronto Empathy Questionnaire (TEQ) [50] 4. The Scale of Perceived Organizational Support [51] 5. Multidimensional Scale of Perceived Social Support [52] 6. Coping Inventory Scale [53]	1. Secondary PTSD	1. History of trauma 2. Low empathy
Beaudoin 2021 [32] Canada	Cross-sectional study	168 primary caregivers of pediatric neurosurgical patients	Person and online survey	Exposure to secondary trauma	1. PTSD Checklist for DSM-5 (PCL-5) [13]	1. PTSD	1. Greater number of surgeries

N/A: Not applicable.

## 4. Discussion

The aim of this systematic review was to estimate the risk of developing secondary PTSD among pediatric health care providers as well as all the additional contributing factors. From the results, it appeared that there was a direct relationship between PTSD and burnout. It is known that hazardous occupations exposed to continuous trauma have an increased risk of developing secondary PTSD [54]. Burnout appears to be a contributing factor in the development of PTSD; therefore, continuous and chronic work stress can be considered to be a predictor of PTSD [55]. The results of this review also showed that pediatric health care providers who worked in intensive care units were more at risk of developing PTSD. In one study [56], the incidence rate of PTSD was 22.38% among ICU nurses as nurses were more prone to psychological trauma due to heavy workloads, sudden emergencies or bullying by colleagues. Another study showed that approximately half of ICU nurses met the criteria for PTSD [57].

In addition, our results showed that nurses were more likely to suffer from PTSD. Dealing with stress has always been part of the nursing job. A survey [58] found that more than 50% of nurses reported feeling exhausted (72%), overwhelmed (64%), irritable (57%) and stressed and could not relax (57%) as a result of their job. According to our results, women were more susceptible to the development of PTSD. According to the literature, women show a higher risk of developing PTSD compared with men due to hormonal changes [59] and childbirth experiences [60].

Anxiety and depression are often co-occurring disorders of PTSD [61]. However, we did not know whether these disorders existed beforehand or were a result. A history of traumatic life events may also indicate an older PTSD. Older traumas can be revived under appropriate conditions and create a new PTSD [62]. Regarding the coping style, it is known that problem-focused coping strategies aim to change or eliminate a stressor [63]; our results confirmed this. Therefore, pediatric health care providers who do not use problem-focused strategies are at a greater risk of stress-related illnesses than those who use emotion-focused strategies [64].

Our results also indicated that empathy plays a catalytic role in the development of PTSD [19,27]. As our results were equivocal, it is important to emphasize that high or low levels of empathy were directly related to PTSD. For example, according to other studies, empathic abilities were reduced in PTSD because there were difficulties in exchanging emotions [65]. Nevertheless, higher levels of empathy—which may demonstrate a greater emotional sensitivity—predispose a person to more severe PTSD symptoms [66]. Finally, alcohol use and PTSD were related. In more detail, people who had drinking problems were more likely to have PTSD; conversely, people with PTSD more often had drinking problems [5].

This study had a few strengths and limitations. A significant strength was the fact that it was the first study to include pediatric professionals of all specialties. The important limitation concerned the design of the studies, which were all cross-sectional. Therefore, we do not know the progression of symptoms over time and the possible interventions that may increase or decrease post-traumatic symptomatology.

## 5. Conclusions

The presence of secondary traumatic stress in health care providers was reported in all studies included in this systematic review. The mental injury caused to pediatric health professionals is special and needs substantial treatment. Health policy-makers should take the specificity in the working environment of the pediatric sector seriously into consideration, especially emergency and PICU departments. We recommend frequent assessments of the mental health of employees as well as more time off for rest, support from mental health specialists and a change of department in cases where the employee wishes it or shows symptoms of mental injury. Further studies are needed in more countries to prospectively assess all risk factors and also to be able to assess post-traumatic symptoms after appropriate interventions. Experimental studies and/or clinical trials are necessary to increase the possibility of conducting meta-analyses. In particular, the special population of nurses that appeared to be affected more than other professional groups should be investigated.

## Figures and Tables

**Table 1:** Methodological quality of the studies [22].

Criteria	Cornille 1999 [23]	Robins 2009 [24]	Meadors 2010 [25]	Czaja 2011 [11]	Sekol 2014 [26]	Berger 2015 [27]	Branch 2015 [28]	Colville 2017 [29]	Kellogg 2018 [30]	Rodríguez-Rey 2019 [31]	Ratrout 2020 [20]	Beaudoin 2021 [32]
1. Research question clearly stated	Yes	Yes	Yes	Yes	Yes	Yes	Yes	Yes	Yes	Yes	Yes	Yes
2.Study population clearly specified and defined	Yes	Yes	Yes	Yes	Yes	Yes	Yes	Yes	Yes	Yes	Yes	Yes
3. Participation rate of eligible people at least 50%	Yes	Yes	Yes	No	Yes	No	Yes	Yes	No	Yes	Yes	Yes
4. Same or similar study population and prespecified inclusion/exclusion criteria	Yes	Yes	Yes	Yes	Yes	Yes	Yes	Yes	Yes	Yes	Yes	Yes
5. Sample size justification	No	Yes	No	No	Yes	No	Yes	Yes	Yes	Yes	Yes	No
6. Exposure to interest measured prior to the outcome	Yes	Yes	Yes	Yes	Yes	Yes	Yes	Yes	Yes	Yes	Yes	Yes
7. Sufficient timeframe between exposure and outcome	N/A	N/A	N/A	N/A	N/A	N/A	N/A	N/A	N/A	N/A	N/A	N/A
8. Different levels of the exposure related to the outcome examined	Yes	Yes	Yes	Yes	Yes	Yes	Yes	Yes	N/A	N/A	N/A	N/A
9. Clearly defined exposure measures	Yes	Yes	Yes	Yes	Yes	Yes	Yes	Yes	Yes	Yes	Yes	Yes
10. Exposure assessed more than once over time	N/A	N/A	N/A	N/A	N/A	N/A	N/A	N/A	N/A	N/A	N/A	N/A
11. Outcome measures clearly defined	Yes	Yes	Yes	Yes	Yes	Yes	Yes	Yes	Yes	Yes	Yes	Yes
12. Outcome assessors blinded to the exposure status	N/A	N/A	N/A	N/A	N/A	N/A	N/A	N/A	N/A	N/A	N/A	N/A
13. Loss to follow-up after baseline of 20% or less	N/A	N/A	N/A	N/A	N/A	N/A	N/A	N/A	N/A	N/A	N/A	N/A
14. Confounding variables measured and statistically adjusted	Yes	Yes	Yes	Yes	Yes	No	Yes	Yes	Yes	Yes	Yes	Yes

Yes: the criterion was met; No: the criterion was not met; N/A: the criterion was not applicable due to the study design.

## Data Availability

Not applicable.
